# Identification of Neoantigens and Construction of Immune Subtypes in Prostate Adenocarcinoma

**DOI:** 10.3389/fgene.2022.886983

**Published:** 2022-04-25

**Authors:** Yukui Gao, Guixin Wang, Yanzhuo Chen, Mingpeng Zhang, Wenlong Gao, Zhiqun Shang, Yuanjie Niu

**Affiliations:** Tianjin Institute of Urology, the Second Hospital of Tianjin Medical University, Tianjin, China

**Keywords:** mRNA vaccine, immunotype, prostate adenocarcinoma, tumor immune microenvironment, immune landscape

## Abstract

**Background:** Messenger ribonucleic acid (mRNA) vaccine has been considered as a potential therapeutic strategy and the next research hotspot, but their efficacy against prostate adenocarcinoma (PRAD) remains undefined. This study aimed to find potential antigens of PRAD for mRNA vaccine development and identify suitable patients for vaccination through immunophenotyping.

**Methods:** Gene expression profiles and clinical information were obtained from TCGA and ICGC. GEPIA2 was used to calculate the prognostic index of the selected antigens. The genetic alterations were compared on cBioPortal and the correlation between potential antigen and immune infiltrating cells was explored by TIMER. ConsensusClusterPlus was used to construct a consistency matrix, and identify the immune subtypes. Graph learning-based dimensional reduction was performed to depict immune landscape. Boruta algorithm and LASSO logistic analysis were used to screen PRAD patients who may benefit from mRNA vaccine.

**Results:** Seven potential tumor antigens selected were signiﬁcantly positively associated with poor prognosis and the antigen-presenting immune cells (APCs) in PRAD, including ADA, FYN, HDC, NFKBIZ, RASSF4, SLC6A3, and UPP1. Five immune subtypes of PRAD were identified by differential molecular, cellular, and clinical characteristics in both cohorts. C3 and C5 had immune “hot” and immunosuppressive phenotype, On the contrary, C1&C2 had immune “cold” phenotype. Finally, the immune landscape characterization showed the immune heterogeneity among patients with PRAD.

**Conclusions:** ADA, FYN, HDC, NFKBIZ, RASSF4, SLC6A3, and UPP1 are potential antigens for mRNA vaccine development against PRAD, and patients in type C1 and C2 are suitable for vaccination.

## Introduction

Prostate adenocarcinoma is the most common cancer and remains the third leading cause of cancer death in men (Global Burden of Disease Cancer Collabration et al., 2017; Litwin and Tan, 2017). Although most men diagnosed with primary prostate adenocarcinoma are treated with surgery, ADT therapy, or radiation therapy, treatments fail in 30% of patients within 10 years resulting in a metastatic disease (Boorjian et al., 2007; Attard et al., 2016; Litwin and Tan, 2017). Other therapeutic strategies, such as checkpoint inhibitors with different immunotherapeutic agents (Abida et al., 2019), hormonal therapy (enzalutamide) (Chen et al., 2019), radiation therapy (radium 223) (Michalski et al., 2018), DNA-damaging agents (Olaparib) (de Bono et al., 2020), or chemotherapy (docetaxel) (Conteduca et al., 2019) also have limited efficacy due to intrinsic chemo- and immune-resistance.

It is well known that the prostate is a non-essential organ with a variety of tumor-associated antigens that can serve as potential targets. In addition, prostate adenocarcinoma is also an inert tumor, which provides sufficient time for the development of antitumor immune responses. Thus, prostate adenocarcinoma is an ideal model for therapeutic cancer vaccines (Bilusic et al., 2017). Recently, a series of mRNA vaccines for PRAD have been introduced into clinical trials. For example, Kubler et al. applied CV9103 to patients with advanced castration-resistant prostate cancer and prolonged overall survival (OS) in a phase I/IIa study, while patients also showed good tolerance and immune response to the vaccine (Kubler et al., 2015). Jinming Li et al. reported that as a therapeutic vaccine, MS2 VLP-based mRNA vaccine delayed tumor growth, demonstrating the efficacy and safety of mRNA vaccine (Li et al., 2014). However, the progress of mRNA vaccine research is slow, and the selection of suitable patients is also a challenge. This makes it an urgent need to explore new PRAD mRNA vaccine antigens and establish a standard for identification of suitable patients.

Our study aimed to find novel PRAD antigens for the development of mRNA vaccine and map the immune landscape of PRAD to select suitable patients for vaccination. Seven candidate genes associated with inferior prognoses and higher infiltration of antigen-presenting cells were identified from PRAD amplified and mutated genes. Subsequently, we defined five robust immune subtypes of PRAD in the TCGA cohort and validated them in the ICGC cohort based on clustering of immune-related genes. Finally, the immune landscape of PRAD was defined by analyzing the distribution of the relevant gene signatures among individual patients. Our findings reveal the different status of the tumor immune microenvironment (TIME) in each PRAD patient and provide a reliable reference for the development of mRNA vaccines.

## Methods

### Data Extraction

Raw counts of RNA-seq data and clinical data of 144 PRAD patients were obtained from International Cancer Genome Consortium (ICGC https://www.icgc-argo.org), and another 499 samples from The Cancer Genome Atlas (TCGA https://www.cancer.gov/tcga). Then the RNA-seq counts were transformed into transcript per million (TPM) to avoid errors in data processing. A total of 2,483 immune-related symbols were retrieved from the Immport Database (Bhattacharya et al., 2018) (https://browser.immport.org/browser). Finally, 2026 immune-related genes were identified for follow-up data analysis after comparison with Req-seq data from the TCGA database.

### GEPIA Analysis

Gene Expression Profiling Interactive Analysis (Tang et al., 2019) (GEPIA2, http://gepia2.cancer-pku.cn, version 2) was used to calculate the differential gene expression and patient survival data. One-way ANOVA was used to identify differentially expressed genes with q values < 0.05 and |log2FC| > 1. The Kaplan-Meier method was used to evaluate OS and PFS, with a cut-off value of 50% (median), and the log-rank test was used for comparison. And adjusted *p*-value < 0.05 was considered statistically significant.

### cBioPortal Exome Analysis in PRAD

We used cBio Cancer Genomics Portal (Cerami et al., 2012) (cBioPortal, http://www.cbioportal.org) to analyze copy number alterations (CNAs), mutations in the raw RNA-seq data from TCGA databases and compare genetic alterations in PRAD.

### TIMER Analysis

The relationship between the abundance of tumor immune infiltrating cells (TIIC) and mRNA vaccine antigens was analyzed on Tumor Immune Estimation Resource (Li et al., 2017) (TIMER, https://cistrome.shinyapps.io/timer/). Spearman correlation analysis was used to select the purity adjustment. *p*-value < 0.05 was considered statistically significant.

### Identification of the Immune Subtypes in PRAD

Clusters were made based on their gene expression profiles of immune cell lineages. The 2026 immune-related genes were clustered and a consistency matrix was established to identify corresponding gene modules and immune subtypes. The partition around medoids (PAM) algorithm was applied with distance measured as ‘1—Pearson’ correlation coefficient, and 500 replicates of bootstraps were performed, each involving 80% patients of the TCGA cohort. K value of cluster varied from 2 to 6 and the optimal partition was determined by evaluating the consensus matrix and the consensus cumulative distribution function. We got consistent results in the ICGC cohort in the same settings. The consistency of immune subtypes between TCGA and ICGC cohorts was quantified by calculating the Pearson correlation between the proportion within the group and the centroid of gene module score.

### Prognostic Evaluation of Immune Subtypes

The log-rank test was used to evaluate the prognostic values of the immune subtypes, and univariate Cox regression analysis was performed with PFS as the endpoint. The association of immune subtypes with different immune-related molecular and cellular characteristics was determined using the Kruskal–Wallis test.

### Establishment of Immune Landscape

To visualize the distribution of immune subtypes across individual patients, the reduceDimension function was used to perform dimension reduction with Monocle implementation of Discriminative Dimension Reduction Tree (DDRTree) algorithm (Qi et al., 2017). The maximum number of components was set to 5. Finally, the immune landscapes were displayed with color-coded immune subtypes using the function plot cell trajectory.

### Construction and Evaluation of Predictors For Suitable Patients Identiﬁcation by Machine Learning Methods

The genes intersecting between LASSO and Boruta analysis were considered the most critical relevant variables for further analysis, which were visualized by a Venn diagram. Then, the discriminative performance of the predictors was evaluated by receiver operating characteristic (ROC) curve analysis, and the optimal cutoff values, AUCs, sensitivity, speciﬁcity, and accuracy were determined.

## Results

### Identification of Candidate Antigens of PRAD

**FIGURE 1:**
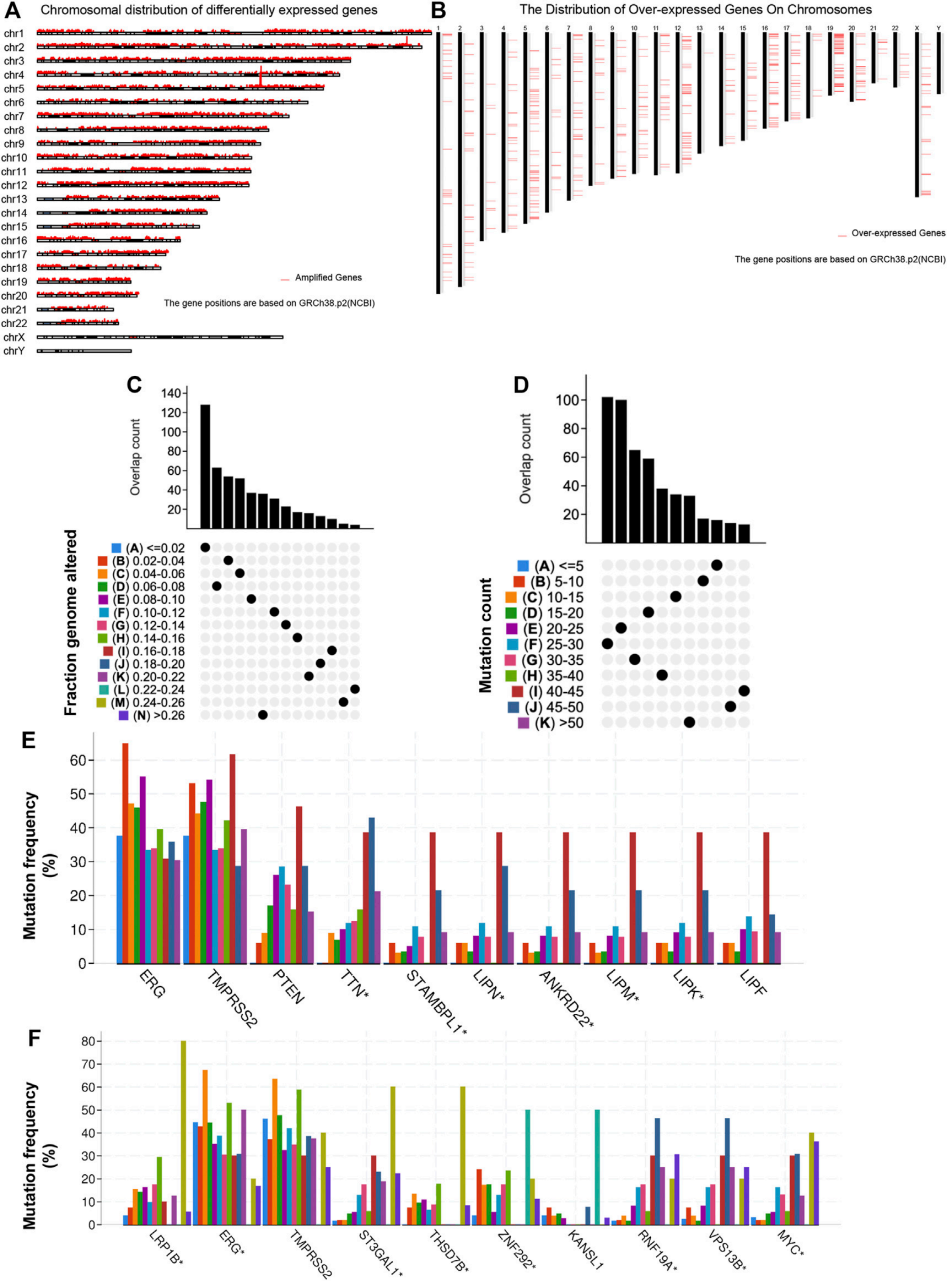
Identification of potential tumor antigens in PRAD. **(A)** The chromosomal distribution of the aberrant copy number genes in PRAD is shown. **(B)** The chromosomal distribution of over-expressed genes in PRAD is shown. **(C–F)** Identification of potential tumor-specific antigens in PRAD. Overlapping mutated genes distributed in the fraction genome altered group **(C)** and mutation count group **(D)** are shown. Genes with the highest frequency in the mutation count groups **(E)** and fraction genome altered groups **(F)** are individually shown.

To identify potential antigens of PRAD, 18,335 amplified genes (Supplementary Table S1) and 3,016 overexpressed genes (Supplementary Table S2) that could express the tumor-associated antigens were explored (Figures 1A,B). Then, a total of 10,402 mutant genes (Supplementary Table S3) potentially encode tumor-specific antigens were screened by evaluating fraction genome alteration and mutation counts in individual samples (Figures 1C,D). Genes with the highest mutation frequency in the mutation count group, including ETS transcription factor ERG, Transmembrane serine protease 2, Phosphatase and Tensin homolog, Titin, STAM binding protein-like 1, Lipase family member N, Lipase family member M, Lipase family member K, Lipase family member F and Ankyrin repeat domain 22, were individually displayed (Figure 1E). Gene with the highest mutation frequency in the fraction genome altered group, including LDL receptor-related protein 1B, ETS transcription factor ERG, Transmembrane serine protease 2, ST3 beta-galactoside alpha-2,3-sialyltransferase 1, thrombospondin type 1 domain containing 7B, Zinc finger protein 292, ring finger protein 19A, RBR E3 ubiquitin-protein ligase, vacuolar protein sorting 13 homolog B and MYC proto-oncogene, were displayed separately (Figure 1F). Consistent with previous reports, the ERG, TMPRSS2, PTEN, and zinc finger protein family are closely related to the occurrence and progression of prostate adenocarcinoma. For example, TMPRSS2-ERG gene fusion often predicts the progression and poor prognosis of prostate adenocarcinoma (Linn et al., 2016). Phosphatase and tensin homolog (PTEN) loss has long been associated with adverse findings in early prostate adenocarcinoma (Jamaspishvili et al., 2020). In total, 1,003 genes with amplification, overexpression, and mutation were identified.

### Identification of Tumor Antigens Associated With PRAD Prognosis and Antigen-Presenting Cells

**FIGURE 2:**
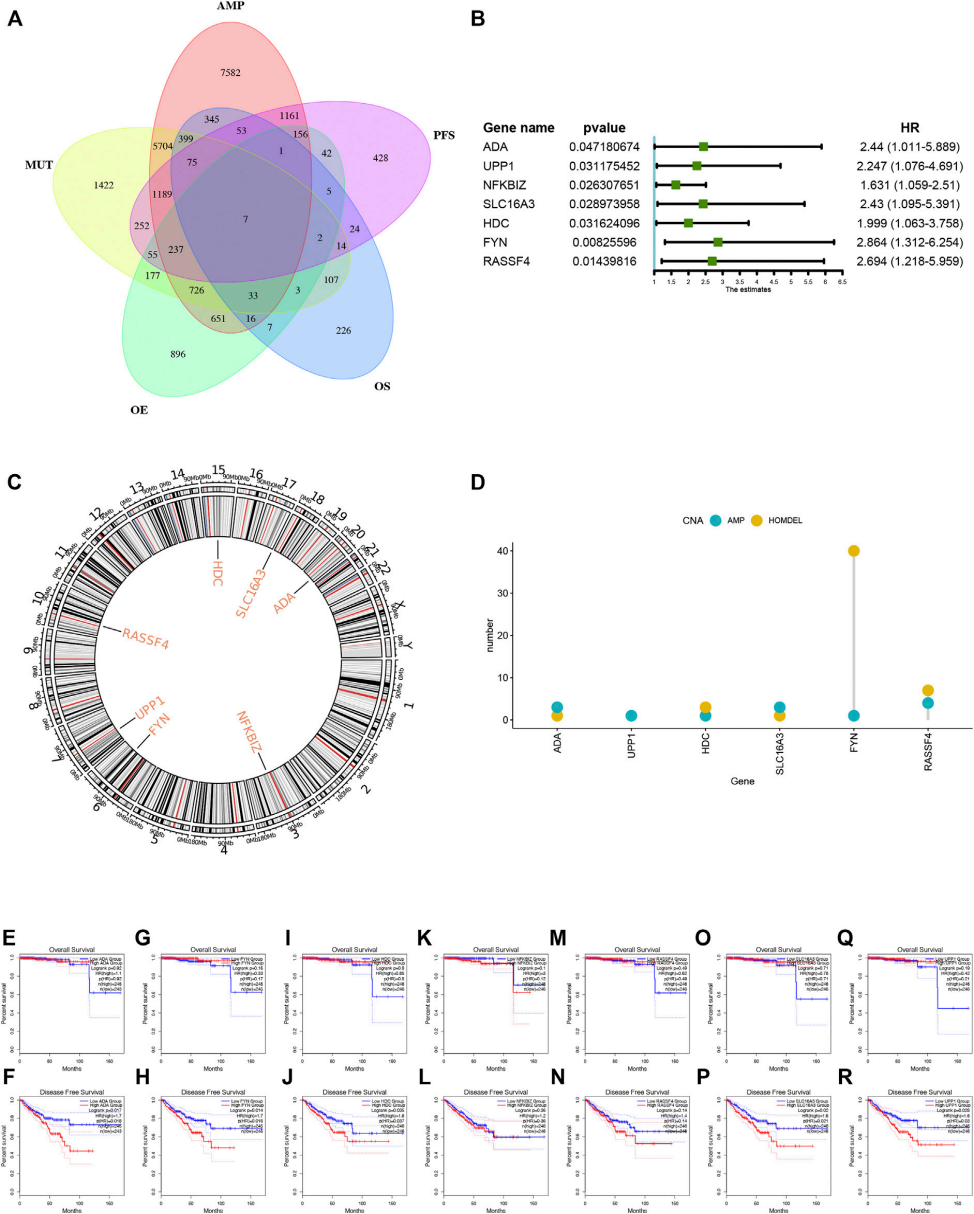
Identification of tumor antigens associated with PRAD prognosis. **(A)** Venn diagram identifying the potential tumor antigens with both amplified and mutated features, and significant OS and PFS prognosis (in a total of 7 candidates) in PRAD. **(B)** Forest maps of single factor survival analysis of 7 genes of PAAD. **(C)** Circos plots of 7 genes revealing the position of chromosomes. **(D)** The amplified and Homdel state of 7 candidate genes. **(E–R)** Kaplan-Meier OS and PFS curves comparing the groups with different expression of ADA **(E–F**), FYN **(G,H)**, HDC **(I,J)**, NFKBIZ **(K,L)**, RASSF4 **(M,N)**, SLC16A3 **(O,P)** and UPP1 **(Q,R)** in PRAD.

**FIGURE 3:**
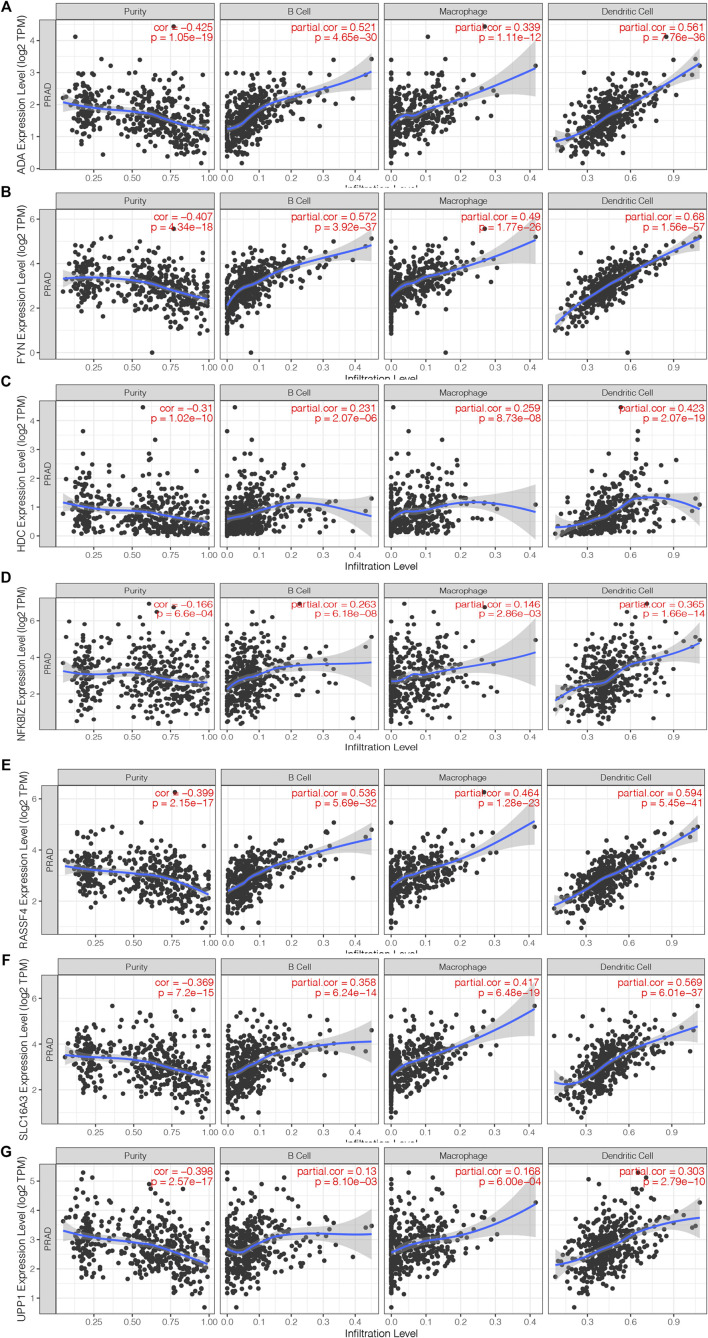
Identification of tumor antigens associated with antigen-presenting cells. **(A–G)** Correlation of 7 candidate genes with antigen presenting cells. Association of ADA **(A)**, FYN **(B)**, HDC **(B)**, NFKBIZ **(D)**, RASSF4 **(E)**, SLC16A3 **(F)** and UPP1 **(G)** expression with the purity of infiltrating cells and amount of macrophages, dendritic cells and B cells in PRAD.

A total of 40 genes associated with the OS of PRAD were identified, of which 7 genes were related to the PFS (Figure 2A). As shown in Figure 2B, the univariate Cox regression analysis showed that the expression of the above 7 genes was related to the prognosis of PRAD patients. Figures 2C,D showed that the chromosomal distribution and genomic variation of 7 genes above-mentioned. The elevated expression of ADA (Figures 2E,F), FYN (Figures 2G,H), HDC (Figures 2I,J), NFKBIZ (Figures 2K,L), RASSF4 (Figures 2M,N), SLC16A3 (Figures 2O,P) and UPP1 (Figures 2Q,R) indicated a worse prognosis of PRAD. Furthermore, the higher expression levels of ADA (Figure 3A), FYN (Figure 3B), HDC (Figure 3C), NFKBIZ (Figure 3D), RASSF4 (Figure 3E), SLC16A3 (Figure 3F), and UPP1 (Figure 3G) were significantly correlated with increased tumor infiltration of macrophages, Dendritic cells (DCs), and B cells, suggesting that the seven tumor antigens may have potential immune-stimulatory effects. In summary, a total of 7 candidate genes with critical roles in PRAD development and progression were identified. These findings indicate that seven tumor antigens (ADA, FYN, HDC, NFKBIZ, RASSF4, SLC6A3, and UPP1) have potential immune-stimulating effects and can be processed and presented by antigen-presenting cells (APC) to induce tumor responses, and are hopeful candidates for developing anti-PRAD mRNA vaccine.

### Construction of Immune Subtypes in PRAD

**FIGURE 4:**
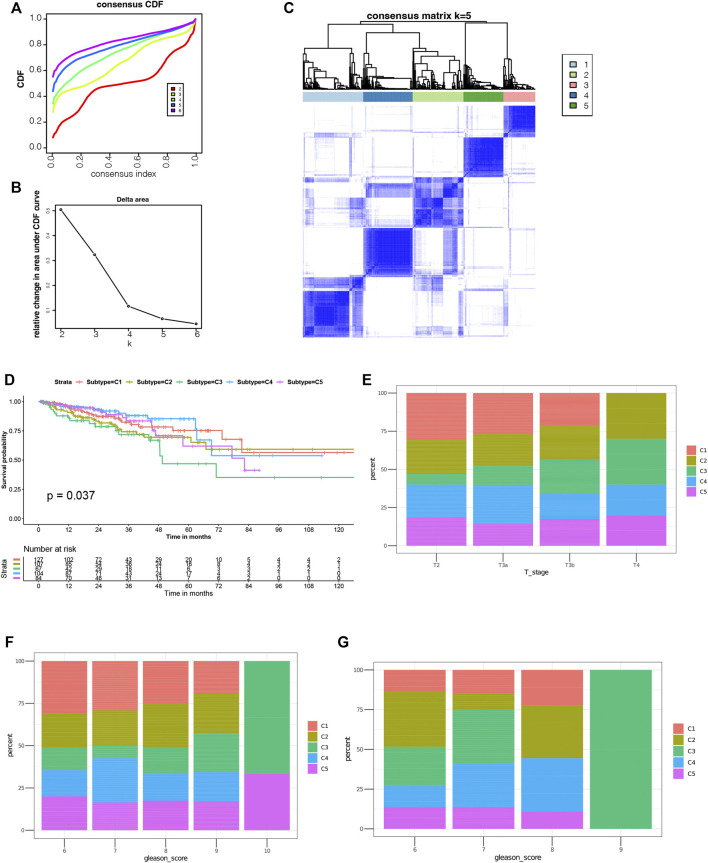
Identification of potential immune subtypes of PRAD. **(A)** Cumulative distribution function curve and **(B)** delta area of immune-related genes in TCGA cohort. **(C)** Sample clustering heat map. **(D)** Kaplan-Meier curves showing prognosis of PRAD immune subtypes in TCGA cohort. **(E,F)** Distribution of C1-C5 across PRAD **(E)** stages and **(F)** Gleason score in TCGA cohort. **g** Distribution ratio of C1-C5 across PRAD Gleason score in ICGC cohort.

**FIGURE 5:**
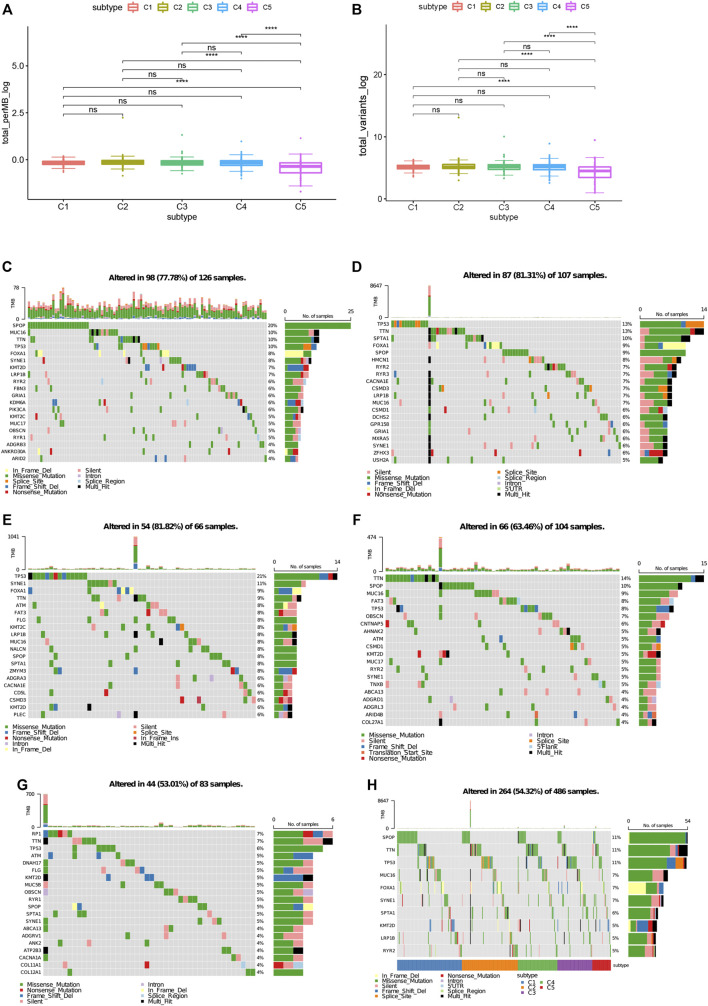
Association between immune subtypes and TMB and mutation. **(A)** TMB and **(B)** mutation number in PRAD C1-C5. **(C)** Top highly mutated genes in PRAD immune subtypes C1. **(D)** Top highly mutated genes in PRAD immune subtypes C2. **(E)** Top highly mutated genes in PRAD immune subtypes C3. **(F)** Top highly mutated genes in PRAD immune subtypes C4. **(G)** Top highly mutated genes in PRAD immune subtypes C5. **(H)** Eleven highly mutated genes in PRAD immune subtypes. *p < 0.01, **p < 0.001, ***p < 0.0001, and ****p < 0.00001.

Through immunotyping, we can recognize the immune status of tumor and its microenvironment, and determine the patients suitable for vaccination. Then we analyzed the expression data of 2026 immune-related genes in 499 PRAD samples from the TCGA cohort to construct consensus clustering. Based on their cumulative distribution function and function δ area, we chosen *k* = 5, where immune-related genes seem to be stably clustered (Figures 4A,B), and 5 immune subtypes designated as C1-C5 were obtained (Figure 4C). C1 and C2 showed a significant correlation with superior prognosis, whereas C3 showed the poorest survival probability (Figure 4D). The distribution of different T stages and Gleason score in subtypes showed that patients diagnosed in different stages were clustered dispersedly. The Gleason score 10 group and stage T4 were significantly related to C3 (Figures 4E,F). Consistent with the results obtained from the TCGA cohort, the Gleason score 10 group also showed a significant correlation with C3 in the ICGC cohort (Figure 4G). In conclusion, immunotyping can be used to predict the prognosis of patients with PRAD, and its accuracy is better than traditional Gleason score and staging, which is consistent across different cohorts.

### Association of Immune Subtypes With TMB and Mutation

Higher tumor mutational burden (TMB) and somatic mutation rates are associated with stronger anticancer immunity (Rooney et al., 2015). Therefore, we calculated the TMB value and mutation for each patient and performed the same analysis in all immune subtypes using the TCGA mutation data set processed by mutect2. C1 and C2 showed much higher TMB and mutated genes counts compared to C5 (Figures 5A,B). Furthermore, Comparing with C5 (53.01%), C1 and C2 had higher mutation rates (81.31%, 81.82%). Surprisingly, C3 and C4 also showed higher mutation rates (81.82%, 63.46%) among the five subtypes (Figures 5C–G). The mutation frequency of 11 famous PRAD related genes including SPOP, TTN, TP53, MUC16, and FOXA1, etc. were the highest in each subtype (Figure 5H). In all, our findings indicate that immunotyping can be used to predict TMB and somatic mutation rates in PRAD patients and that patients in type C1 and C2 with higher TMB and mutated genes counts may have positive feedback on mRNA vaccines.

### Association of Immune Subtypes of PRAD With ICPs and ICD

**FIGURE 6:**
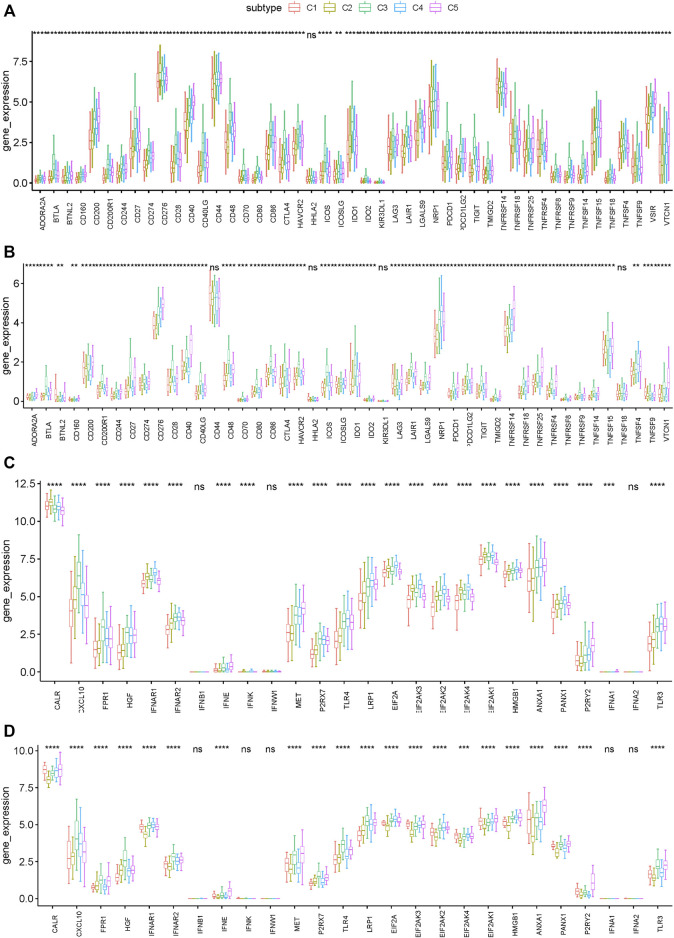
Association between immune subtypes and ICPs and ICD modulators. **(A,B)** Differential expression of ICP genes among the PRAD immune subtypes in **(A)** TCGA and **(B)** ICGC cohorts. **(C,D)** Differential expression of ICD modulator genes among the PRAD immune subtypes in **(C)** TCGA and **(D)** ICGC cohorts. *p < 0.01, **p < 0.001, ***p < 0.0001, and ****p < 0.00001.

Immune checkpoints (ICPs) and immunogenic cell death (ICD) modulators play critical roles in regulating host anti-tumor immunity. We then analyzed their expression levels in different immune subtypes. A total of 47 ICPs related genes were analyzed in TCGA and ICGC cohorts, of which 46 (97.9%) genes in the TCGA cohort (Figure 6A) and 43 (91.5%) in the ICGC cohort (Figure 6B) had differential expression between the immune subtypes. For example, CD200, CD200R1, CD244, CD27, CD28, CD40, CD40LG, CD48, CD80, CD86, CTLA4, HAVCR2 and ICOS, etc. were overexpressed in the C3 and C5 tumors in the TCGA cohort, which is consistent in the ICGC cohort.

In addition, the general expression level of ICPs in the TCGA cohort was higher than that in the ICGC cohort. 26 ICD related genes were detected in the TCGA cohort, of which 23 (88.5%) had differential expression between the immune subtypes (Figure 6C). Similarly, 26 ICD related genes were detected in the ICGC cohort, of which 21 (80.8%) had differential expression between the immune subtypes (Figure 6D). For example, CALR, IFNAR1, EIF2AK3, EIF2AK2, EIF2AK4, and EIF2AK1 showed higher expression levels in C1 and C2 tumors in both cohorts. Collectively, immunotyping can mirror the expression level of ICD modulators and ICPs, and be used as potential therapeutic biomarkers for mRNA vaccines. According to the above results, C3 and C5 were more likely to show immunosuppressive phenotype. By comparison, patients with C1 and C2 are suitable for mRNA vaccine.

### Association of Immune Subtypes With Tumor Markers

**FIGURE 7:**
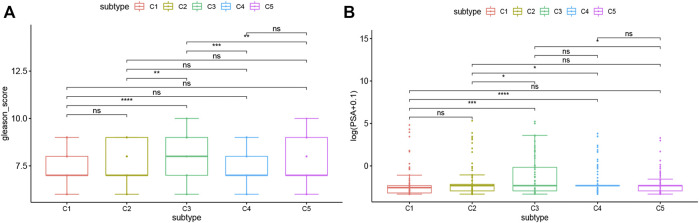
Association between immune subtypes and Gleason score and serum PSA level. **(A)** Gleason score in PRAD immune subtypes in TCGA cohorts. **(B)** serum PSA level in PRAD immune subtypes in TCGA cohorts.

Gleason score and serum PSA level are currently recognized indicators for the prognosis and diagnosis of PRAD. The higher levels of them often indicate the progress, poor prognosis, or recurrence of PRAD. In this study, the levels of Gleason score and serum PSA were analyzed for each patient in TCGA and ICGC cohorts. Both TCGA and ICGC cohorts showed significant differences in Gleason score and serum PSA level among the immune subtypes. For example, compared with C3 and C5, C1 and C2 had significantly lower Gleason score and serum PSA level in the TCGA cohort (Figures 7A,B). These results were consistent with the superior prognosis showed in the C1 and C2 patients. It is also confirmed that Gleason score and serum PSA level are important prognostic markers of PRAD. Taken together, in predicting the prognosis of PRAD patients, the immune subtype is not inferior to the Gleason score and serum PSA level.

### Cellular and Molecular Characteristics of Immune Subtypes

**FIGURE 8:**
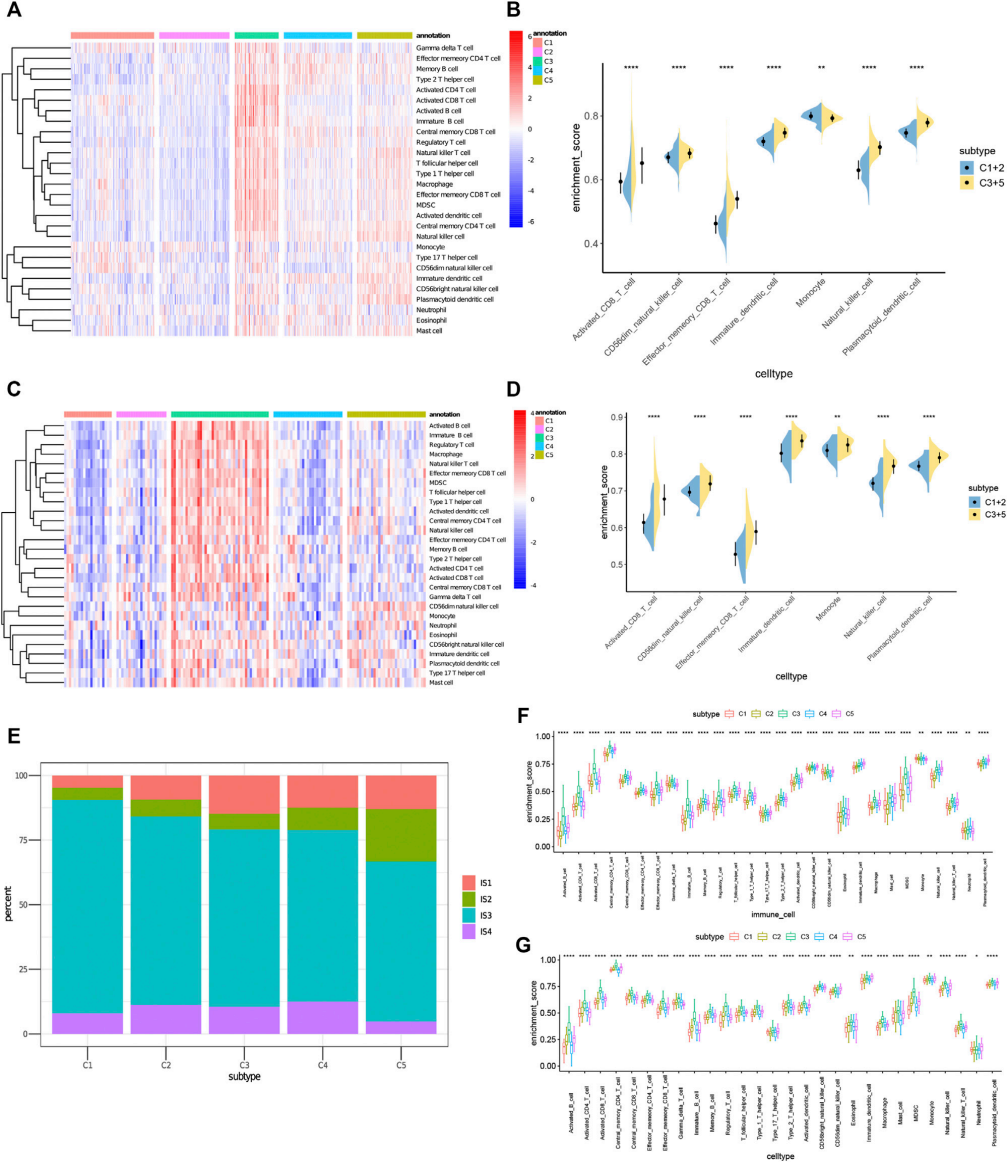
Cellular and molecular characteristics of immune subtypes. **(A,C)** Differential enrichment scores of 28 immune cell signatures among PRAD immune subtypes in **(A)** TCGA and **(C)** ICGC cohorts. **(B,D)** Differential enrichment scores of 7 prognostically relevant immune cell signatures in **(B)** TCGA and **(D)** ICGC cohorts. **(E)** Overlap of PRAD immune subtypes with 4 pan-cancer immune subtypes IS1-IS4. **(F,G)** Differential enrichment scores of 28 immune signatures among PRAD immune subtypes in **(F)** TCGA and **(G)** ICGC cohorts.

The effectiveness of mRNA vaccines largely depends on the immune status of the tumor. Therefore, we used ssGSEA to score 28 previously reported characteristic genes in the TCGA and ICGC cohorts to define immune cell components in the five immune subtypes. As shown in Figure 8A, the immune cell components were divided into 5 subtypes. There were significant differences in immune cell composition among different subtypes. Almost all the immune cell related genes mentioned in C3 and C5 groups were significantly higher than those in C1 and C2 groups in TCGA cohorts (Figures 8A,B). Thus, C3 and C5 were immune “hot” and immunosuppressive phenotypes, while C1 and C2 were immune “cold” phenotypes. A similar trend occurred in the ICGC cohort, as well (Figures 8C,D). Thus, these results indicate that the immune subtype can mirror the PRAD immune status, and identify patients suitable for mRNA vaccination. The induction of immune responses in patients with immune “cold” C1 and C2 tumors treated with the mRNA vaccine seems promising.

To prove the reliability of this immune classification, we next explored the correlation between the five immune subtypes and the six previously reported immune classifications (Thorsson et al., 2018). For the convenience of clear description, they were named IS1-IS6. Of which PRAD was mostly clustered into IS3. C1 and C2 mainly overlapped with IS3 and IS4, C3 with IS3 and IS1, and C5 with IS1, IS2 and IS3 (Figure 8E). IS3 was associated with superior and worse prognoses respectively, IS1 and IS2 indicated intermediate prognoses. These findings were consistent with the observed longer survival of patients with C1and C2 tumors than those with C3 and C5 tumors. Surprisingly, C1 patients with superior prognosis and C3 patients with the poorest survival largely overlapped with IS3. These results not only proved the reliability of our immunotyping method but also augmented the previous classification. We also tried to obtain 28 immune cell markers from the public database and used the ssGSEA algorithm to calculate the immune cell infiltration rate in TCGA and ICGC cohorts. The correlation between the immune subtypes and identified 28 significantly associated immune cells, as well as 17 associated immune signatures in the TCGA cohort, were analyzed (Charoentong et al., 2017). As shown in Figures 8F,G, C3 and C5 had the most obvious immune characteristics and much higher immune cell infiltration rates in almost all groups. Therefore, C3 and C5 were associated with an overall favorable immune-activated phenotype and characterized by diverse immune signatures. In contrast, the lower scores of immune signatures in C1 and C2 were indicative of an immune ‘cold’ phenotype. To sum up, immune subtypes can reflect the cellular and molecular characteristics of patients with PRAD and also indicate their immune status. Therefore, immune subtypes are hopeful biomarkers for mRNA vaccines. Patients without the immunosuppressive microenvironment, such as immune “cold” C1 and C2 tumors, may be suitable candidates for mRNA vaccination.

### The Immune Landscape of PRAD

**FIGURE 9:**
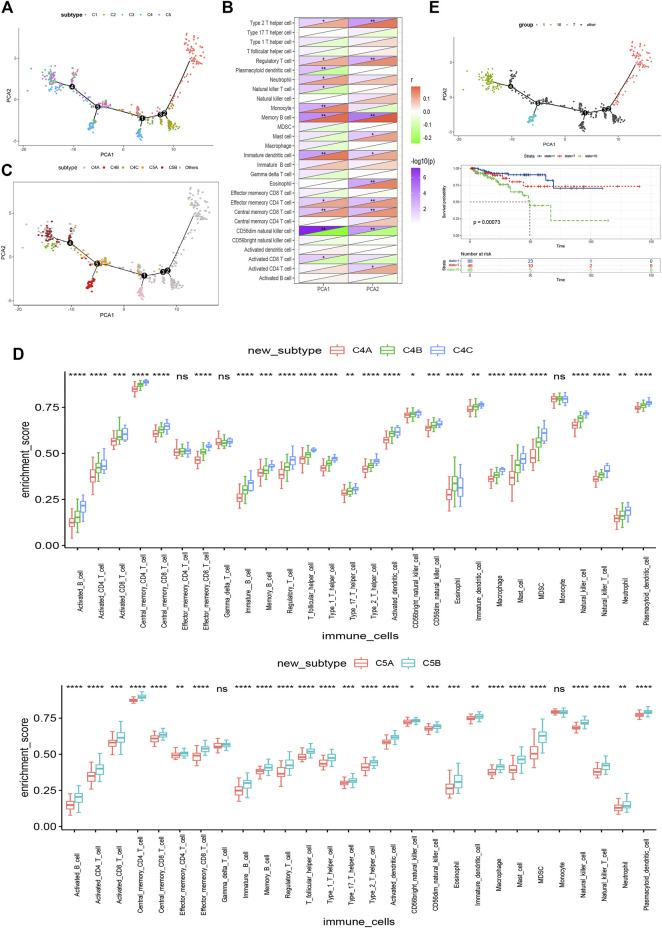
Immune landscape of PRAD. **(A)** Immune landscape of PRAD. each point represents a patient and the immune subtypes are color-coded. The horizontal axis represents the first principal component and the vertical axis represents the second principal component. **(B)** Heat map of two principal components with 28 immune cell signatures. **(C)** Immune landscape of the subsets of PRAD immune subtypes. **(D)** Differential enrichment scores of 28 immune cell signatures in the above subsets. **(E)** Immune landscape of samples from three extreme locations and **(F)** their prognostic status.

In this study, we constructed the immune landscape of PRAD using the immune gene expression profiles of individual patients (Figure 9A). The *X*-axis correlated with a variety of immune cells, of which plasmacytoid dendritic cells and CD56^dim^ natural killer cells showed the most negative correlation, and the *Y*-axis was most negatively correlated with CD56^dim^ natural killer cells (Figure 9B). The integral distribution of C3 and C5 was opposite to that of C1 and C2. Surprisingly, even the same subtypes show the opposite distribution, which indicates significant intra-cluster heterogeneity within the subtypes, especially in C4 and C5. C4 was further divided into three subsets and C5 into two subsets according to the location of the immune cell population (Figure 9C). There were significant differences in the enrichment scores of several immune cells between the subtypes. (Figure 9D). For instance, C4A and C5A had lower counts of activated B cells, activated CD4 + T cells, activated CD8 + T cells, effector memory CD8 + T cells, regulatory T cells, and bone marrow-derived suppressor cells (MDSC). Therefore, mRNA vaccines may be relatively feasible and more effective in C4A and C5A. In addition, after comparing the prognosis of samples with extreme distribution in the immune landscape, we found that group 1 showed the best survival probability. This result is consistent with the above results (Figures 9E,F). In conclusion, these findings suggest that the immune landscape based on immune subtypes can accurately identify the immune components of each PRAD patient and predict their prognoses, which is helpful to select the appropriate patients for mRNA vaccine.

### Construction and Evaluation of Predictors For Suitable Patients Identiﬁcation

**FIGURE 10:**
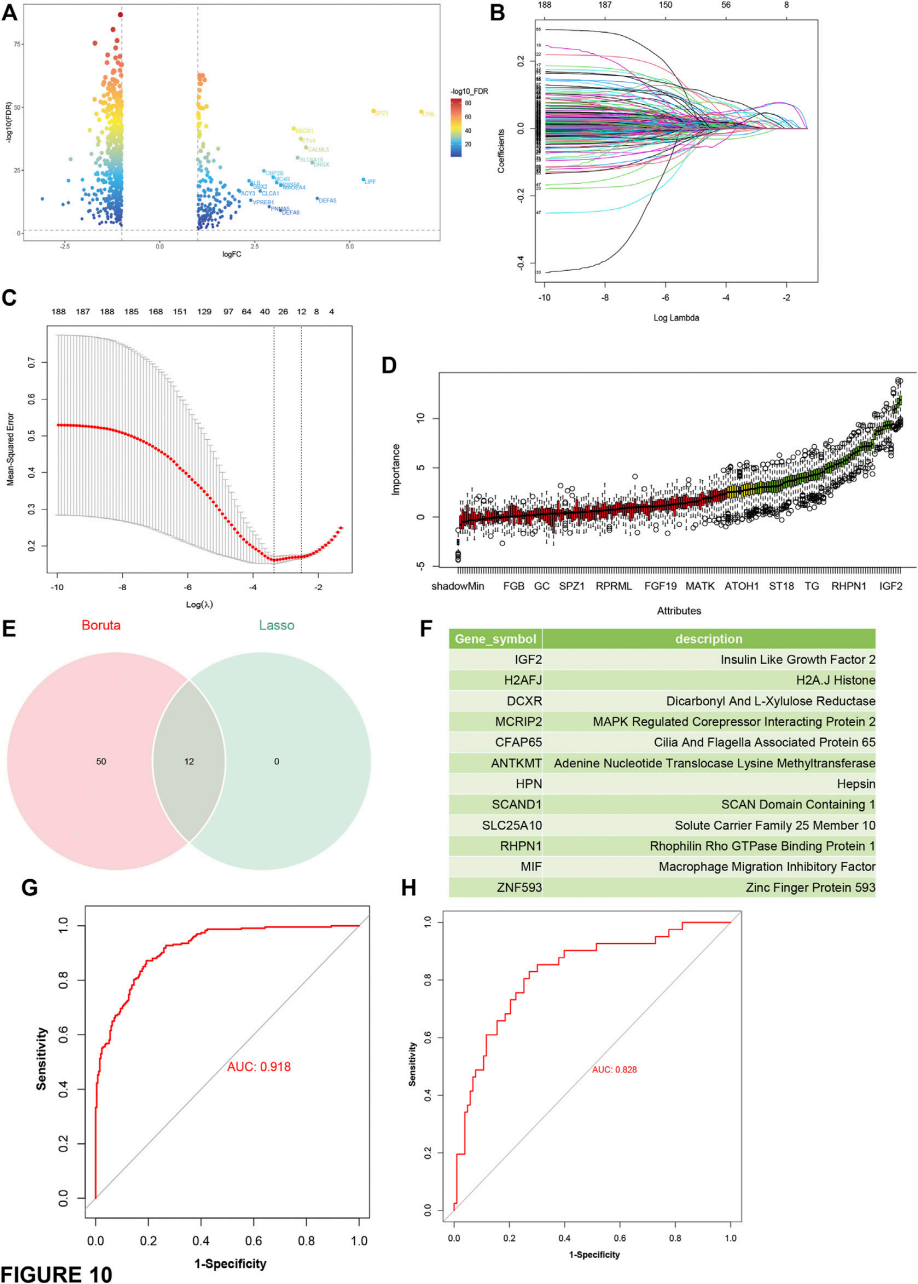
Construction and evaluation of predictors based on the most representative genes of C1 and C2 subtype. **(A)** Volcano plot compares gene expressions between C1 and C2 and other subtypes. Genes with log2 (fold-change) beyond 1 with adjusted *p*-value (FDR) lower than 0.05 were considered as significantly upregulated in C1 and C2 subtype **(B)** LASSO regression analysis: coefﬁcient values at varying levels of penalty. Each curve represents a gene. **(C)** Ten-fold cross-validation was used to calculate the best lambda, contributing to the minimum mean cross-validated error (cvm). **(D)** Boruta analysis: Importance plot of the genes. Green boxes represent important features (retained), and red boxes represent unimportant features (declined). **(E)** Venn diagram identifying the most critical C1 and C2 speciﬁc variables that were shared by the LASSO and Boruta methods. **(F)** list of the most representative genes of C1 and C2. **(G,H)** ROC curves of predictors for distinguishing C1 and C2 subtype and other subtypes in the training cohort (TCGA) **(G)** and the test cohort (ICGC) **(H)**.

Then, differential gene expression analyses were performed between C1 and C2 and other groups (Figure 10A). The most representative genes of C1 and C2 were used to identify immune “cold” tumor-related features using machine learning methods. A total of 12 genes (Supplementary Table S4) and 62 genes (Supplementary Table S5) were identiﬁed by least absolute shrinkage and selection operator (LASSO), binomial deviance, and Boruta analyses (Figures 10B–D) respectively, and the 12 genes shared by both methods were determined as the speciﬁc features of C1 and C2 tumors (Figure 10F). In addition, the C1 and C2 predictor genes also had an AUC of 0.919 and 0.828 in the TCGA cohort and the ICGC cohort (Figures 10G,H), respectively. Taken together, the gene-set of 12 predictors (IGF2, H2AFJ, DCXR, MCRIP2, CFAP65, ANTKMT, HPN, SCAND1, SLC25A10, RHPN1, MIF, and ZNF593) has an excellent performance in the identification of patients suitable for mRNA vaccine.

## Discussion

PRAD is the most common cancer in humans and the second leading cause of cancer deaths in men in the United States each year (Penning, 2015). For patients with advanced or metastatic prostate adenocarcinoma, the main treatment is androgen-deprived therapy (ADT). These patients who relapse after ADT treatment are called castration-resistant prostate adenocarcinoma patients. Despite the second-generation antiandrogen providing temporary respite, resistance to ADT therapy occurs frequently (Liu et al., 2014). In recent years, immunotherapy has made rapid progress. It has revolutionized oncology from the therapeutic perspective, but its effectiveness in PRAD is still unclear (Zappasodi et al., 2018; Liu et al., 2020).

In our study, the profiles of PRAD somatic mutations and amplified genes were constructed, which revealed a wide range of potent antigen targets that might be considered in PRAD. Since the antigens predicted using the gene alteration profile might not be functionally significant in PRAD, prognostic roles and immune correlations were further analyzed to confirm the clinical relevance of the selected antigens. Seven tumor antigens (ADA, FYN, HDC, NFKBIZ, RASSF4, SLC6A3, and UPP1) associated with inferior prognoses and higher-level infiltration of antigen-presenting cells in PRAD were identified, thus promising candidates for mRNA vaccine. Their upregulation is associated with high APC and B cell infiltration, which indicates strong immunogenicity. Thus, these antigens play a key role in the development and progression of PRAD and can be directly processed and presented to CD8 + T cells to induce immune attacks with sufficient lymphocyte infiltration. Although further clinical evaluation is needed, the potential of the seven tumor antigens to become successful targets for anti-PRAD mRNA vaccines has been consolidated in previous reports. For example, Yun Qu et al. have reported that the overexpression of ADA could enhance the antitumor efﬁcacy of CART cells which has been proved as an effective immunotherapy for several tumors (Qu et al., 2021). Also, Daniela Zanini et al. demonstrated that ADA activity had poor prognostic significance in lung adenocarcinoma (Zanini et al., 2019). All these suggest that ADA has good immunogenicity and can effectively activate immune responses. Although the immunogenicity of FYN has not been experimentally proven, its prognostic impact on a variety of tumors is obvious, such as breast cancer (Xie et al., 2016), pancreatic cancer (Dong et al., 2020). Histidine decarboxylase (HDC), the histamine producing enzyme, is commonly induced at inﬂammatory sites during the late and chronic phases of both allergic and non-allergic inﬂammation (Hirasawa, 2019). Kamil Slowikowski et al. found that NFKBIZ, as key regulators of inflammation in synovial fibroblasts, mediates leukocyte recruitment by regulating production of multiple cytokines and chemokines (Slowikowski et al., 2020). According to the review of Liu Aimei et al., RASSF4 plays a dual role in tumors (Liu et al., 2022). Lei Xue et al. found that SLC16A3 has good prognostic significance in lung adenocarcinoma (Xue et al., 2021). Recently, UPP1 was reported to play a vital role in immune and inflammatory biological process during particular events such as chronic atrophic gastritis (Yang et al., 2020) and respiratory allergy (Remy et al., 2014).

Since mRNA vaccine is only beneficial for a fraction of cancer patients, Stratification of PRAD patients based on tumor immune-related gene profiles was performed. We divide PRAD patients into five immune subtypes to select the right population for vaccination. Five immune subtypes showed different molecular, cellular, and clinical characteristics. In the TCGA and ICGC cohorts, patients with type C1&C2 had better prognosis than other subtypes. This suggests that immunophenotyping can be used to predict the prognosis of PRAD patients, and we demonstrated that immunophenotyping has higher predictive accuracy compared with established tumor markers such as Gleason score and serum PSA level and traditional staging. In addition to predicting prognosis, immunotyping can also indicate therapeutic response to mRNA vaccines. Compared with C3 and C5, patients in type C1 and C2 tumors with higher TMB and somatic mutation rates may have positive feedback on mRNA vaccine. The high expression of ICPs in C3 and C5 tumors in the TCGA and ICGC cohort indicates an immunosuppressive tumor microenvironment, which may suppress the mRNA vaccine to trigger an effective immune response. On the contrary, the expression of ICD modulators was increased in C1 and C2 tumors across both cohorts, suggesting that mRNA vaccine has greater potential in these immune subtypes. In addition, the complex immune environment of PRAD indicates that there is considerable heterogeneity among individual patients and within the same immune subtype, which also suggests that we should further study mRNA vaccines suitable for personalized treatment.

Since tumor immune status is a determinant of vaccine immune efficacy, we then investigated the immune cell components of different subtypes. These findings showed that C3 and C5 were immune ‘hot’ phenotypes, while C1 and C2 were immune ‘cold’ phenotypes. Combined with the above results, we found that the molecular characteristics of these tumors are consistent with the immune characteristics, indicating that the response of patients with different immune subtypes to mRNA vaccine will be significantly different. For example, C1 and C2 were significantly associated with low expression of CD8^+^ T cells, lymphocytes and stromal fractions, and TGF-β-responsive gene signatures, suggesting an immune “cold” phenotype. To avoid poor immunogenicity of these tumors, an mRNA vaccine that triggers immune cell infiltration to stimulate the immune system may be an appropriate choice. Thus, in C1 and C2, combining mRNA vaccines with ICB or ICD modulators may rejuvenate the immune system and increases immune cell infiltration.

According to previous immunotyping studies, PRAD was divided into IS1-IS4 subtypes. Most patients were clustered into the IS1, IS2, IS3, and IS4 subtypes. IS3 was associated with better prognosis, IS1 and IS2 were associated with moderate prognosis, and IS4 was associated with poorer prognosis. In this study, PRAD was divided into C1-C5 subtypes. C1 and C2 mainly overlapped with IS3 and IS4, C3 with IS3 and IS1, and C5 with IS1, IS2 and IS3. These results were consistent with the better survival probability of C1 and C2, and the relatively poor prognoses of C3 and C5. Interestingly, both C1 with a better prognosis and C3 with the lowest survival mostly overlapped with IS3. Thus, our immunophenotyping method is reliable and complementary to previous classifications. Furthermore, we carried out the differential gene analysis of C1 and C2 compared to other groups, then Lasso and Boruta analysis was performed to identify the predictors of patients with C1 and C2 tumors.

Obviously, bioinformatics data analysis is only the first step in the research, and prostate cancer neoantigens are meaningful only if it is verified in clinic. We will carry out in-depth exploration at the clinical level to further confirm the role of mRNA vaccine and after enough experiments are accumulated, human-level research will be carried out, which will open up new research directions for improving the treatment effect of PRAD.

## Conclusion

ADA, FYN, HDC, NFKBIZ, RASSF4, SLC6A3, and UPP1 are promising PRAD antigens for developing mRNA vaccines. Patients with immune subtypes C1 and C2 are suitable candidates for vaccination. Our findings provide a theoretical basis for anti-PRAD mRNA vaccine development, predicting patient prognosis, and screening patients suitable for vaccination.

## Data Availability

The datasets presented in this study can be found in online repositories. The names of the repository/repositories and accession number(s) can be found in the article/Supplementary Material.

## Supplementary Material

Supplementary Table S1 The amplified genes of PRAD in TCGA database.

Supplementary Table S2 The overexpressed genes of PRAD in TCGA database.

Supplementary Table S3 The mutant genes of PRAD in TCGA database.

Supplementary Table S4 The 12 representative genes identified by LASSO algorithm between C1 and C2 with other groups.

Supplementary Table S5 The 62 representative genes identified by Boruta analyses between C1 and C2 with other groups.

## Abbreviations

APC, Antigen presenting cells; DC, Dendritic cells; ICPs, Immune checkpoints; ICB, Immune checkpoint blockage; ICD, Immunogenic cell death; ICGC, International Cancer Genome Consortium; MDSCs, Myeloid-derived suppressor cells; OS, Overall survival; PFS, Progression-free survival; PRAD, Prostate adenocarcinoma; ssGSEA, Single-sample GSEA; TIICs, Tumor infiltrating immune cells; TMB, Tumor mutation burden; TCGA, The Cancer Genome Atlas; TIME, Tumor immune microenvironment.
